# P-1804. Evaluating Implementation Outcomes of Stewardship Interventions for Rapid Blood Culture Diagnostics

**DOI:** 10.1093/ofid/ofae631.1967

**Published:** 2025-01-29

**Authors:** Monica Abdelnour, Christine Lockowitz, Matthew Sattler, Evan E Facer, Jason G Newland

**Affiliations:** Washington University, St. Louis, Missouri; St. Louis Children's Hospital, St. Louis, Missouri; Washington University in St. Louis School of Medicine, Department of Pediatrics, Division of Infectious Diseases, Saint Louis, Missouri; St. Louis Children's Hospital, St. Louis, Missouri; Washington University in St. Louis School of Medicine, St. Louis, Missouri

## Abstract

**Background:**

Rapid diagnostic testing for bloodstream infections, when coupled with antimicrobial stewardship (AS) intervention, has demonstrated improved clinical outcomes. Yet, data regarding implementation outcomes and the necessary strategies for these interventions are limited.

**Table 1:**
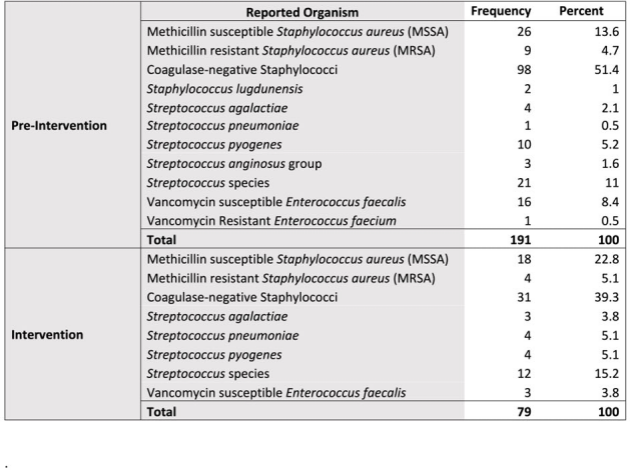
Verigene Gram Positive Blood Culture Nucleic Acid Test (BC-GP) results Pre-intervention and During the Intervention

Frequency and percentage of BC-GP results during the pre-intervention phase and intervention phases are reported. Coagulase negative staphylococci includes Staphylococcus species, Staphylococcus epidermidis, methicillin susceptible Staphylococcus epidermidis, and methicillin resistant Staphylococcus epidermidis BC-GP results.

**Methods:**

A pre-post quasi-experimental design was used to compare children with positive blood culture results. Pre-intervention causative organisms were identified using the Verigene Gram Positive Blood Culture Nucleic Acid Test (BC-GP) and reported to clinicians by a microbiology technologist. Post-intervention organisms were identified and reported similarly, in addition to 24 hours a day/7 days a week (24/7) AS intervention. AS members received BC-GP notifications and communicated antibiotic recommendations to the primary team. Hospital stakeholders were identified in the pre-intervention phase to assess divisional needs. The primary outcome was time to targeted therapy (TTT), calculated as time from BC-GP result to order of predetermined narrowest antibiotic regimen or discontinuation of therapy. TTT was compared by time-series analyses. Secondary outcomes included acceptability, appropriateness, and feasibility, assessed using validated surveys completed by pediatric infectious diseases (ID) clinicians and non-ID clinicians who received the AS intervention.

Proportion of Patients Receiving Targeted Antibiotic Therapy Over Time

**Figure:**
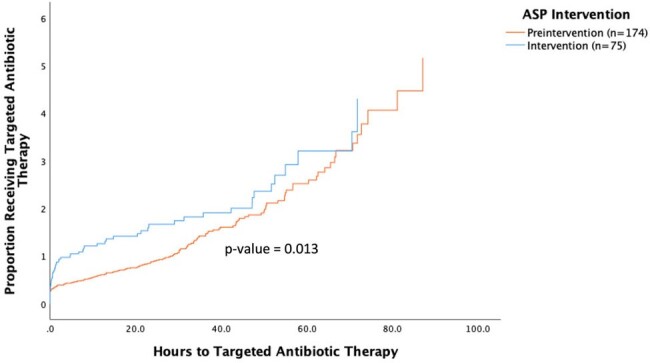

A Kaplan-Meier analysis with a hazard function for time to targeted therapy (TTT) in children with positive blood cultures. Cases in the intervention phase (11/2023-04/2024) are represented in blue, and preintervention (10/2022-10/2023) controls are represented in orange. Censored cases included patients who did not receive targeted antibiotic therapy within their hospital admission. Log rank test demonstrated a statistically significant reduction in TTT in the intervention group versus the pre-intervention group, with a larger proportion of patients in the intervention group receiving targeted antibiotic therapy more rapidly than patients in the pre-intervention group (P-value of 0.013).

**Results:**

The median TTT decreased from 15.9 hours before intervention to 0.9 hours with AS intervention (P=0.013). Validated surveys were completed by 23 ID and 38 non-ID clinicians. Over 80% of ID and non-ID clinicians agreed or strongly agreed that the intervention was acceptable and appropriate. However, significant differences were noted in all feasibility domains, including “doable,” “easy to apply,” “implementable,” and “possible" measures. Specifically, 39% of ID clinicians reported the intervention was “easy” or “very easy” to apply, as compared to 84% of non-ID clinicians (P < 0.0001).

**Figure:**
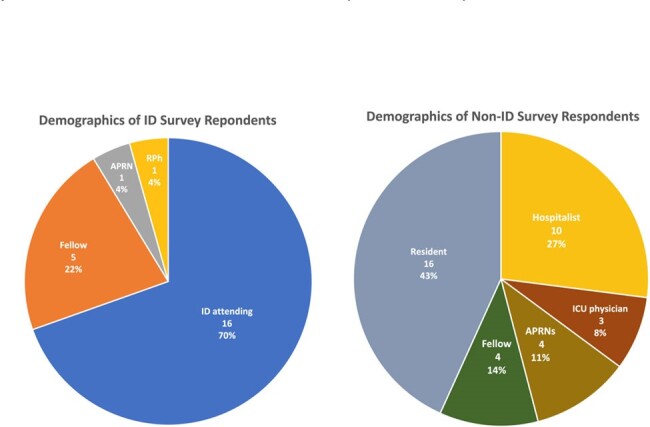

Infectious diseases (ID) respondents categorized by their role within the medical team. APRNs: Advanced Practice Registered Nurses, RPh: Registered Pharmacist.

Non-infectious diseases (ID) respondents categorized by their role within the medical team. APRNs: Advanced Practice Registered Nurses, ICU: Intensive Care Unit.

**Conclusion:**

24/7 AS intervention paired with rapid blood culture diagnostics is associated with reduced TTT and is deemed appropriate and acceptable by ID and non-ID clinicians. The difference in feasibility perception by ID and non-ID clinicians highlights implementation challenges, and warrants evaluation of future strategies.

**Figure:**
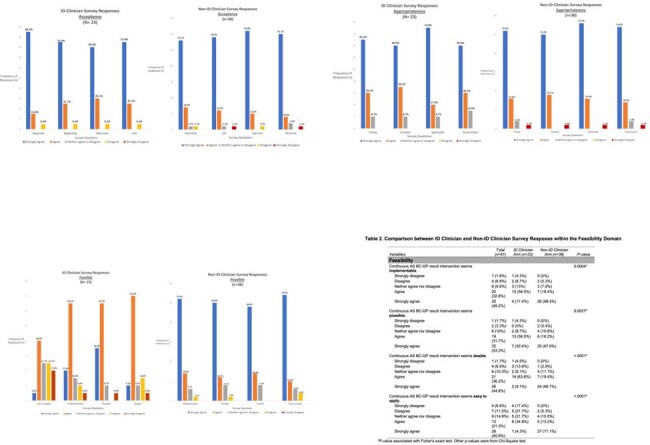

Frequency and percentage of responses from infectious diseases (ID) clinicians in comparison to non-ID clinicians for the three domains of successful implementation outcomes including 1) acceptability, 2) appropriateness, and 3) feasibility. Agreement on the acceptability and appropriateness for the AS intervention was observed among ID and non-ID clinicians with no significant difference between the two groups. Statistically significant differences were noted among responses between ID and non-ID clinicians in the feasibility domain.

Table 2: Frequency and percentage of responses on a Likert scale from ID clinicians (n=23) in comparison to non-ID clinicians (n=38) in the feasibility domain. Chi-Square or Fisher's Exact tests demonstrated significant differences in responses when comparing ID to non-ID clinician’s perception of “doable”, “easy to apply”, “implementable”, and “possible” measures for AS interventions on the BC-GP.

**Disclosures:**

**Jason G. Newland, MD, MEd**, Moderna: Grant/Research Support|Pfizer: Grant/Research Support

